# Complete Genome Sequence of *Tomato Leaf Curl New Delhi Virus* from Luffa in Indonesia

**DOI:** 10.1128/MRA.01605-18

**Published:** 2019-04-11

**Authors:** Fariha Wilisiani, Yutaro Neriya, Mai Tagami, Misaki Kaneko, Sedyo Hartono, Hisashi Nishigawa, Tomohide Natsuaki

**Affiliations:** a Department of Biological Production Science, United Graduate School of Agricultural Science, Tokyo University of Agriculture and Technology, Fuchu, Tokyo, Japan; b Department of Agrobiology and Bioresources, School of Agriculture, Utsunomiya University, Utsunomiya, Tochigi, Japan; c Sano High School, Sano, Tochigi, Japan; d Tochigi Shonan High School, Tochigi, Tochigi, Japan; e Department of Crop Protection, Faculty of Agriculture, Gadjah Mada University, Yogyakarta, Indonesia; KU Leuven

## Abstract

This is the first report of a begomovirus infecting luffa in Indonesia. The genome of this virus shares a close identity with that of *Tomato leaf curl New Delhi virus* (ToLCNDV). There is a 36-nucleotide duplicated sequence in the DNA-B component, suggesting the occurrence of an intraviral recombination.

## Abstract (precis)

This is the first report of a begomovirus infecting luffa in Indonesia. The genome of this virus shares a close identity with that of *Tomato leaf curl New Delhi virus* (ToLCNDV).

## ANNOUNCEMENT

Most begomoviruses (family *Geminiviridae*, genus *Begomovirus*) possess bipartite, circular, single-stranded DNA genomes. Begomoviruses are transmitted by whitefly and cause typical mosaic, yellowing, curling symptoms on leaves and stunted growth of plants resulting in serious damage to crop production in tropical and subtropical regions. Recently, *Tomato leaf curl New Delhi virus* (ToLCNDV) has spread widely in Central and Southeast Asia and Europe and has become one of the emerging begomoviruses. ToLCNDV has a wide host range, which is one of its hallmarks (1).

In August 2017, we collected luffa (*Luffa* sp.) leaves showing yellowing, which is typical for begomovirus infections in Java, Indonesia. As there were no reports of begomovirus infection in luffa in Indonesia at that time, we attempted to identify the potential begomovirus from that sample.

Total DNA was extracted from collected luffa leaves using the DNeasy plant minikit (Qiagen, Germany), followed by PCR amplification (KOD-Plus-Neo, Toyobo, Japan) of a part of the DNA-A components of the begomovirus genome, using universal primers (UPV1 and PAV1c715; product size, 1,621 nucleotides [nt]) (2). Amplified fragments were cloned into the pUC19 vector (TaKaRa Bio, Japan), and at least 3 independent plasmids were sequenced using the BigDye terminator sequencing kit and the Applied Biosystems 3500 genetic analyzer (Thermo Fisher Scientific, USA) with vector-specific primers (5′-GTAAAACGACGGCCAG-3′ and 5′-CAGGAAACAGCTATGACC-3′). Low-quality bases were trimmed at medium stringency from sequence data and then assembled using SeqMan Pro ver. 15.3.0 (DNAStar, USA). We designed a primer set (5′-CTGGACAAACAGGCCGATGAACAG-3′ and 5′-ACCTCCTTCTGAGGTTTATGCGTC-3′, 1,656 nt) for inverse PCR to clone the rest of the genome based on the determined sequence and to amplify the circular DNA fragment covering the complete nucleotide sequence of the genome and sequenced it in the same manner. Homology search analysis using BLASTn indicated that this virus shares high similarity with ToLCNDV isolates. To detect DNA-B, we designed two primer sets based on published ToLCNDV sequences (5′-GGARATCTGYGAAACWCAGSAGG-3′ and 5′-GTKCCVATTAATGCWGTTGGTC-3′, 1,353 nt; 5′-TGGTGGTCGGAATTCGACGTCAGT-3′ and 5′-TGTTTGGGGAGCTTCCGTCATGAC-3′, 1,852 nt). These products were used for cloning and sequencing. Nucleotide sequence identities were calculated using Genetyx-Mac ver. 19 (Genetyx, Japan). No beta-satellites were detected using universal primers (3).

The sequence analysis showed that DNA-A (2,739 nt) and DNA-B (2,724 nt) were predicted to have six and two open reading frames (ORFs), respectively, which is typical for bipartite Old World begomoviruses as determined by ORFfinder (https://www.ncbi.nlm.nih.gov/orffinder/). From homology search analysis using the full genome sequence of DNA-A and DNA-B, this isolate shared high sequence identity with ToLCNDV isolates (Table 1).

**TABLE 1:** Similarity analysis of Indonesian luffa isolate with ToLCNDV isolates

Isolate	GenBank accession no.		Sequence identity (%)	
	DNA-A	DNA-B	DNA-A	DNA-B
ToLCNDV-IN[BD:Tha:01:37:Tom:09]	KM383736	—^*a*^	94.7	—
ToLCNDV-[TW:Mel:07]	GU180095	GU180096	94.5	88.2
ToLCNDV-[ID:JV:Cuc:08]	AB613825	AB613826	94.4	91.8
ToLCNDV-[TH:Luf]	AF102276	—	94.4	—
ToLCNDV-[IN:Son:Luf:05]	AY939926	AY939924	94.0	81.9
ToLCNDV-[PK:Mul:Luf:04]	AM292302	—	92.6	—

a —, no sequence data were found in the DDBJ/ENA/GenBank database.

Although we determined that the length of DNA-B is 2,724 nt, most ToLCNDVs are around 2,690 nt. We found that 36 nt regions between 1258 to 1293 and 1291 to 1326 shared a 91.7% sequence identity, suggesting a duplication of this region. This duplication was located in an untranslated region of DNA-B, and no other known ToLCNDV isolates have this duplication. Currently, it is unknown what the biological significance of this duplication might be.

According to the species demarcation criteria of the genus *Begomovirus* (4), this isolate was classified as one of the ToLCNDV isolates. This is, therefore, the first report of ToLCNDV infecting luffa in Indonesia, and we propose to name this isolate ToLCNDV-[Indonesia:Java:Luffa:2017] (ToLCNDV-[IN:JV:Luf:17]).

### Data availability.

The genome sequences of ToLCNDV-[IN:JV:Luf:17] were deposited at the DDBJ/ENA/GenBank database under accession numbers LC431619 (DNA-A) and LC431620 (DNA-B).
